# Molecular mechanisms in the pathogenesis of sepsis 

**Published:** 2014

**Authors:** V Pop-Began, V Păunescu, V Grigorean, D Pop-Began, C Popescu

**Affiliations:** * ”Carol Davila” University of Medicine and Pharmacy, Bucharest; Department of General Surgery, “Bagdasar – Arseni” Clinical Emergency Hospital, Bucharest; ** Department of Public Health and Health Management, ”Carol Davila” University of Medicine and Pharmacy, Bucharest

**Keywords:** sepsis, molecular mechanisms, microRNA

## Abstract

Innate immune system is a universal form of host defense against infections. The recognition of the innate immunity is based on a limited number of encoded receptors that have evolved to recognize microbial metabolism products. The recognition of these molecular structures allows the immune system to distinguish its own infectious components from non-communicable structures. The immune suppression is a hallmark of sepsis. The complement system is activated in the early stages of sepsis, generating large amounts of anaphylatoxin C5a. Complement and TLRs (toll-like receptors) family are two major upstream sensors and effectors systems of innate immunity. It was found that TLR4 and complement system are involved in the initiation of the inflammatory response in sepsis. Clinical studies in which TLR4 was blocked have not shown beneficial effects. TLRs, that are a subfamily of PRRs (pattern recognition receptors), have emerged as the crucial receptors for the recognition of DAMPs (Damage-associated molecular pattern molecules). Recently, a special form of non-coding genetic material called microRNA has been highlighted in the complex cascade of sepsis. The individual role of every microRNA and the exact role of microRNA network are under investigation. Currently, studies are performed in order to find micro RNA to be used as biomarkers of sepsis. Researches are performed to determine microRNA, small fragments of non-coding RNA, in order to distinguish between patients with sepsis and healthy patients, and if the plasma levels of microRNA correlate with the severity of the disease. Recent researches report that the regulation of gene expression through microRNA plays a very important role in the following cellular processes, for example: apoptosis, the differentiation process, and the cell cycle.

Sepsis is the clinical syndrome defined by the presence of infection and systemic inflammatory response to infection [**1**] and the results from a complex interaction between the host and infectious agents, characterized by the activation of multiple inflammatory pathways, with an increased risk of mortality [**1**,**2**]. Sepsis causes cellular and metabolic changes, with the onset of septic shock with positive blood cultures (sepsis-17%, severe sepsis-25%, septic shock-69%) [**3**] or multiple organ dysfunctions, which are responsible for most part of the deaths [**2**]. Patients admitted with S.I.R.S. (Systemic Inflammatory Response Syndrome) have a long-term hospitalization and an increased mortality [**4**]. Sepsis is a continuous process and a complex clinical syndrome, systemic response to infection, which starts from a self-limited inflammatory response, and if exceeded compensating control mechanisms, often, microbes leave the located area to invade the bloodstream [**1**,**2**,**5**]. Sepsis is the culmination of complex interactions between microorganisms and the host [**5**]. Severe sepsis is sepsis complicated by organ dysfunction, hypoperfusion, or acute alteration in mental status [**3**,**5**]. Septic shock is called infectious shock or toxicoseptic, bacterial shock or bacteremia [**3**-**5**,**9**] and is the most severe complication of sepsis with a considerable mortality [**2**,**4**]. If compensatory anti-inflammatory response is ineffective, multiple organ dysfunction (“Multiple Organ Dysfunction Syndrome”-MODS) is developing. Normal homeostatic mechanisms cannot be maintained without a therapeutic intervention [**4**,**5**]. Multiple organ failure must include one or more of the following conditions: cardiovascular disorders, respiratory, neurological, hepatic, hematologic system disorders and other organ dysfunction [**4**,**6**,**7**]. SIRS is the adaptive response of the systemic homeostasis to the challenges that threaten the patient's life and the organ dysfunction mirrors the consequences of ineffective adaptation [**4**,**5**].

Sepsis is a heterogeneous, dynamic syndrome, caused by the imbalances in the “inflammatory network” [**1**,**6**,**7**]. The microorganisms involved in the pathogenesis of sepsis are Gram-positive bacteria-cocci (staphylococci, streptococci) and Gram-negative bacteria-bacilli (Klebsiella, Pseudomonas aeruginosa, E. coli), fungi (Candida), parasites, viruses. Among bacteria, the host’s immune response, inflammatory and coagulation pathways may be complex interactions [**8**]. Research suggests that fungal pathogenesis is caused by such fungi, as well as an inadequate response of the host, which leads to changes caused by the immune cells. The understanding of the molecular mechanisms of induction of sepsis, triggered by Candida albicans, by identifying the fungal genes that are responsible for specific responses of host organs was proposed [**8**,**10**].

The malfunction of the regulatory mechanisms during sepsis may lead to the loss of control of inflammation due to the excessive activation of the inflammatory response (**Fig. 1**). The attenuation of the systemic inflammatory response induced by sepsis and the homeostatic imbalance occur when C3 and CD14 are inhibited. The reduction of inflammation induced by E. coli suggests a potential therapeutic modality for the treatment of sepsis [**11**].

**Fig. 1:**
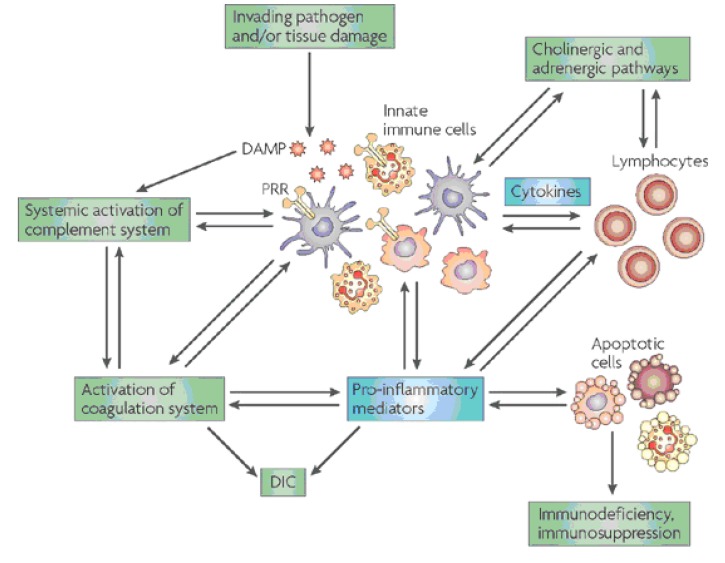
Harmful molecular mechanisms in sepsis (Daniel Rittirsch, Michael A. Flierl & Peter A. Ward, Nature Reviews Immunology (8), october 2008, pp. 776-787)

Legend:

D.A.M.P – Damage-Associated Molecular Pattern (molecules)

P.R.R. – Pattern Recognition Receptors 

D.I.C. – Disseminated Intravascular Coagulation (Syndrome)

Recently, a special form of non-coding genetic material, called microR.N.A., was highlighted in the complex network of sepsis [**12**]. MicroR.N.A. fragments fulfill its function by regulating their target genes, thus directly affecting their expression at the posttranscriptional level, and the connected network of protein-protein interactions (protein-protein interaction network – P.I.N.) [**13**]. A fundamental principle states that the aberrant microR.N.A. can adjust the disease progression according to the biological processes [**14**]. If a fragment of microR.N.A. can be useful as a marker in the diagnosis of sepsis, the biological function of P.I.N., set by this fragment, will largely depend on the development of sepsis. In order to demonstrate the function of controlling the microR.N.A. fragments, in the critical biological processes in sepsis, the ontological analysis of gene for the microR.N.A. fragments that adjusts the P.I.N. has been carried out [**15**].

Abnormal levels of the following types of microR.N.A.: miR-132, miR-146, miR-150 and miR-155 were determined in patients with sepsis. Research is ongoing in order to determine the role of the individual microR.N.A. and the exact role of microR.N.A. network [**16**]. The increase of certain types of microR.N.A.: miR-125a, miR-132, miR-146a, miR-155, miR-9 has been determined in the development of the innate immune response, as a result of stimulation of participating cells (macrophages and monocytes) with LPS (lipopolysaccharides) [**16**]. 

The recent research suggests that the adjustment of the gene expression through microR.N.A. fulfills a very important role in the following cellular processes: apoptosis, cell differentiation and cell cycle [**15**,**16**]. Currently, studies are performed in order to find the microR.N.A. to be used as a biomarker of sepsis. The progress of genetics and molecular biology is related to the expectations of improving the vital prognosis of septic patients. Further, research is carried out in order to determine microR.N.A., small fragments of non-coding R.N.A. in order to distinguish between patients with sepsis and healthy patients and if the plasma levels of microR.N.A. correlate with the severity of the disease [**17**,**18**].

Recently, the T.L.R. family has been recognized as a component of the innate immune system [**16**]. The research performed in vivo reveals that the regulation of Toll-like receptor-mediated inflammatory response is realized by complement [**20**]. Toll-like receptor stimulation by microbial products leads to the activation of antimicrobial genes and signals the inflammatory cytokines [**19**,**20**]. Sepsis is triggered when microbial products activate Toll-like receptors, causing multiple organ failure and subsequent shock and death. The latest findings found that not only microbial substances but also endogenous molecules could stimulate Toll-like receptors [**20**]. CD14 is a pattern of recognition receptor. The inhibition of the complement and CD 14 attenuates the E. coli induced inflammation and may be used as a treatment regimen in Gram-negative sepsis, together with an appropriate antibiotic therapy. In previous preclinical studies conducted by researchers it has been demonstrated that the orientation of the C3 and C5 key molecules of the complement and CD14 of T.L.R. family had a broad anti-inflammatory effect on Gram-negative bacteria [**11**].

During sepsis, C5a, the complement anaphylatoxin is generated by following the activation of the complement system and by the C5-convertase activity of thrombin of the coagulation cascade. C5a triggers the release of pro-inflammatory mediators, including macrophage migration-inhibitory factor (MIF) and high-mobility group box 1 protein (HMGB1), and it activates the coagulation cascade by inducing tissue-factor expression (not shown). HMGB1 is a pleiotropic cytokine that binds to Toll-like receptor 4 (TLR4) and acts as an endogenous alarmin to increase the release of pro-inflammatory mediators. TLR4-mediated responses, in turn, are negatively regulated by C5a. Similar to HMGB1, large amounts of MIF are released during sepsis, which promotes a pro-inflammatory response by amplifying cytokine secretion through the upregulation of TLR4 expression. MIF, which is produced by the pituitary gland as well as by leukocytes, inhibits the anti-inflammatory effects of endogenous glucocorticoids of the endocrine system, which, in turn, induce MIF secretion. HMGB1 links the immune response with the autonomic nervous system, which regulates the release of HMGB1 and other cytokines during sepsis. Interleukin-17A (IL-17A), which is an important regulator of inflammation at the interface between innate and adaptive immunity, orchestrates responses of both innate and adaptive immune cells (**Fig. 2**) [**21**].

**Fig. 2:**
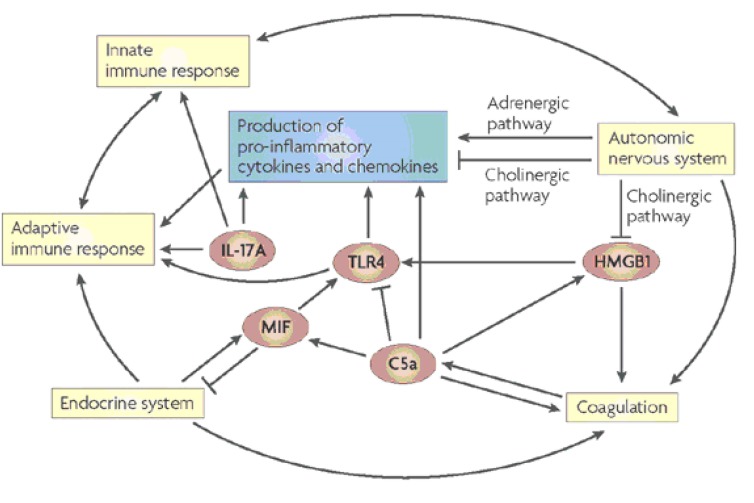
Central hubs of the inflammatory response in sepsis (Daniel Rittirsch, Michael A. Flierl, Peter A. Ward, Harmful molecular mechanisms in sepsis, Nature Reviews Immunology 8, 776-787).

In sepsis, the pro-inflammatory environment causes mononuclear cells to upregulate the expression of tissue factor on their cell surface, leading to the systemic activation of coagulation [**22**]. The coagulation cascade is initiated by the exposure of coagulation factors in the blood to the subendothelial proteins following damage to the blood-vessel endothelium. In primary haemostasis, circulating platelets bind to collagen through their cell-surface glycoprotein Ia/IIa receptors to form a haemostatic plug at the site of injury. The adhesion of platelets is stabilized by large, multimeric von-Willebrand-factor proteins, which form links between platelets, glycoproteins and collagen fibrils. Simultaneously, the action of a complex cascade of coagulation factors (a group of serine proteases that are activated in a sequential manner) results in the formation of fibrin strands, which further strengthen the platelet plug (secondary haemostasis). Traditionally, the coagulation cascade has been described as two pathways: the contact-dependent (intrinsic) activation pathway and the tissue-factor (extrinsic) pathway, the latter being the main pathway for the initiation of blood coagulation. These two pathways converge on the activation of thrombin, which converts fibrinogen to fibrin and ultimately results in the formation of a fibrin-crosslinked clot. The contemporary description of physiological haemostasis in vivo does not divide coagulation into cellular and plasmatic components or different activation pathways, but instead describes that coagulation involves three phases [**22**].

Most patients die in late stages, although there are deaths during the initial phase. The study of the factors involved in sepsis will allow the development of new therapeutic modalities. Sepsis is a source of increased morbidity and mortality [**1**,**2**,**5**]. 
